# Congenital heart disease and chromossomopathies detected by the karyotype

**DOI:** 10.1590/0103-0582201432213213

**Published:** 2014-06

**Authors:** Patrícia Trevisan, Rafael Fabiano M. Rosa, Dayane Bohn Koshiyama, Tatiana Diehl Zen, Giorgio Adriano Paskulin, Paulo Ricardo G. Zen

**Affiliations:** 1 UFCSPA, Porto Alegre, RS, Brasil; 2 Hospital Materno Infantil Presidente Vargas (HMIPV), Porto Alegre, RS, Brasil

**Keywords:** heart defects, congenital, karyotype, Down syndrome, trisomy, chromosome aberrations

## Abstract

**OBJECTIVE:**

To review the relationship between congenital heart defects and chromosomal abnormalities detected by the karyotype.

**DATA SOURCES:**

Scientific articles were searched in MEDLINE database, using the descriptors "karyotype" OR "chromosomal" OR "chromosome" AND "heart defects, congenital". The research was limited to articles published in English from 1980 on.

**DATA SYNTHESIS:**

Congenital heart disease is characterized by an etiologically heterogeneous and not well understood group of lesions. Several researchers have evaluated the presence of chromosomal abnormalities detected by the karyotype in patients with congenital heart disease. However, most of the articles were retrospective studies developed in Europe and only some of the studied patients had a karyotype exam. In this review, only one study was conducted in Latin America, in Brazil. It is known that chromosomal abnormalities are frequent, being present in about one in every ten patients with congenital heart disease. Among the karyotype alterations in these patients, the most important is the trisomy 21 (Down syndrome). These patients often have associated extra-cardiac malformations, with a higher risk of morbidity and mortality, which makes heart surgery even more risky.

**CONCLUSIONS:**

Despite all the progress made in recent decades in the field of cytogenetic, the karyotype remains an essential tool in order to evaluate patients with congenital heart disease. The detailed dysmorphological physical examination is of great importance to indicate the need of a karyotype.

## Abstract (es)

**OBJETIVO:**

Realizar una revisión de la literatura sobre la relación de las cardiopatías congénitas con anormalidades cromosómicas detectadas por medio del examen de cariotipo.

**FUENTES DE DATOS:**

Se investigaron artículos científicos en el portal MEDLINE, utilizándose los descriptores "*karyotype*", OR "*chromosomal*" OR "*chromosome*" AND "*heart defects, congenital*". La investigación se limitó a artículos publicados en inglés a partir de la década de los 1980.

**SÍNTESIS DE LOS DATOS:**

Las cardiopatías congénitas son un grupo de lesiones etiológicamente heterogéneo y poco comprendido. Varios investigadores evaluaron la presencia de anormalidades cromosómicas detectadas por el examen de cariotipo en pacientes portadores de cardiopatía congénita. Sin embargo, la mayoría de los artículos estaba compuesta por trabajos retrospectivos desarrollados en Europa, en los que no todos los pacientes fueron sometidos a la evaluación cariotípica. En esta revisión, se encontró solamente un estudio desarrollado en Latinoamérica, en Brasil. Se sabe que anormalidades cromosómicas son frecuentes, estando presentes en aproximadamente uno a cada diez pacientes con cardiopatía congénita. Entre las alteraciones observadas, se destaca la trisomía del cromosoma 21 (síndrome de Down). Esos pacientes frecuentemente presentan malformaciones extracardíacas asociadas y riesgo más grande de morbidez y mortalidad, lo que hace la cirugía cardíaca todavía más arriesgada.

**CONCLUSIONES:**

A pesar de todos avances ocurridos las últimas décadas en el área de la citogenética, el examen de cariotipo sigue siendo una herramienta fundamental para evaluar pacientes con cardiopatía congénita. El examen físico dismorfológico minucioso, realizado por un pediatra experimentado o por un geneticista, es de gran importancia para indicar la realización del examen.

## Introduction

Congenital malformations are detected in approximately 3 to 5% of newborns^(^ 1 ^)^, and one in every 33 presents severe abnormalities^(^ 2 ^)^. Major malformations are those that cause an adverse effect on the social acceptability of the individual or in the functioning of a determined organ or system^(^ 3 ^)^. On the other hand, minor malformations do not present aesthetical or functional significance for the individual, being a structural finding that occurs in less than 4% of the general population. However, some minor anomalies may be external markers of more specific anomalies, sometimes hidden. Therefore, most syndromes could be recognized by the clinical geneticist if these patterns of minor anomalies were taken into consideration. The dysmorphology assessment, therefore, could help support the decision on whether to perform a complementary investigation, such as, for instance, through karyotyping^(^ 4 ^)^. 

Among the most frequent congenital malformations, congenital heart defects stand out, comprising structural and functional heart abnormalities present at birth, regardless of the time of diagnosis. Congenital heart defects are a heterogeneous group of lesions with varying hemodynamic consequences, requiring different follow-ups and interventions^(^ 5 ^)^. Studies show that the incidence of congenital heart disease can vary from four to 14 per 1,000 live births^(^ 6 ^-^ 8 ^)^. In Brazil, studies described a prevalence that ranges from five to 12 per 1,000 live births^(^ 2 ^,^ 9 ^,^ 10 ^)^. These variations can be explained by several factors, such as the occurrence of lethal defects that prevent a live birth of the fetus and the exclusion of minor cardiac defects. Studies have shown that congenital heart disease may be responsible for about 40% of all birth defects, and it is considered one of the most frequent malformations^(^ 11 ^,^ 12 ^)^. In Brazil, despite its great geographical extent, there are 12 specialized centers both in the diagnosis and in the treatment of patients with congenital heart defects^(^ 13 ^)^. The average number of cardiovascular surgeries at birth in Brazil is of approximately 23,077 procedures per year. However, the current health network is not enough for the demand and in 2002, for instance, there was a surgery deficit that reached 65%^(^ 7 ^)^. 

Thus, congenital heart defects are an even greater public heath problem worldwide, being the leading cause of death among congenital malformations^(^ 12 ^)^. Severe and moderately severe heart defects account for about three to six out of every 1,000 live births and are characterized by the need for more intensive and complex surgical care^(^ 6 ^,^ 11 ^,^ 14 ^)^. These defects are a major cause of admission and mortality in pediatric intensive care units^(^ 15 ^)^. In Rio Grande do Sul, however, most intensive care units are overcrowded and often lack equipment and skilled professionals for the differential diagnosis as well as conditions for the surgical treatment of patients with congenital heart disease^(^ 10 ^)^. This may be due to the fact that less developed countries have other priorities related to health, including the preventions of malnutrition and promoting vaccination campaigns^(^ 13 ^)^.

The heart is the first organ to form in the embryo, and it is vital for the provision of oxygen and nutrients to the developing fetus^(^ 8 ^)^. Its formation is complex and occurs over several weeks of the embryonic life, making it very vulnerable to the occurrence of failures during its development^(^ 15 ^)^. Congenital heart defects are considered etiological heterogenic malformations and are poorly understood^(^ 16 ^,^ 17 ^)^. Only around 15-20% of cases are attributed to known causes^(^ 5 ^,^ 18 ^)^ and, among them, chromosomal abnormalities^(17,19) ^stand out, which are more frequent in patients with congenital heart defects than in the general population^(^ 16 ^,^ 20 ^,^ 21 ^)^. 

The first steps towards the development of the karyotype began with the understanding of the action of colchicine and the hypotonic treatment of the cells, which occurred in the first half of the 20th century. The determination of the correct number of chromosomes in human somatic cells (n=46) by Tjio and Levan, in 1959^(^ 22 ^)^, was the basis for identifying chromosomal syndromes. In 1959, Lejeune et al described the first trisomy of autosomal chromosomes in a case of Down syndrome^(^ 23 ^)^. Some decades later, the introduction of techniques for longitudinal staining of chromosomes, known as "banding"^(^ 24 ^)^, and the emergence of techniques for high chromosomal resolution^(^ 25 ^)^ allowed the numerical and structural chromosomal changes to be better recognized and diagnosed. As seen by its historical outline, the karyotype test is regarded as a nearly handmade examination, based on cell culture (usually blood), which has numerous steps and, therefore, it is potentially subject to faults (due, for instance, to the form of material collection), besides presenting a long duration (the results are usually obtained only a few weeks after sample collection)^(^ 26 ^)^.

With the development of DNA probes and the techniques of fluorescence in situ hybridization - FISH, spectral karyotyping (SKY), and comparative genomic hybridization (CGH), from the 1980s, a new concept was created, that of molecular cytogenetics^(^ 26 ^,^ 27 ^)^. These new techniques allowed the identification of complex and very subtle changes, such as very small deletions and duplications (microdeletions and microduplications, respectively), which may not appear in a standard cytogenetic analysis by karyotyping. Another advantage over the karyotype test is that many of these techniques may not require cell culture for analysis, which enables faster results^(^ 26 ^,^ 28 ^)^. These techniques have a high cost, higher than that of the karyotype, but their implementation has allowed the identification of new conditions, such as the 22q11 deletion syndrome, also known as velocardiofacial or DiGeorge syndrome, a genetic disorder closely related to congenital heart disease which, most often, escapes detection by karyotyping^(^ 29 ^-^ 32 ^)^. 

However, despite all advances, the karyotype, even with its limitations, remains as a fundamental tool in the genetic evaluation of patients, including those with congenital heart disease. The karyotype applies mainly to those patients with minor anomalies or other major extracardiac changes. As seen, these can be markers of conditions that are often hidden, such as some syndromes. Hence the importance of the patients' dysmorphology assessment to better select the cases to be tested. In Rio Grande do Sul, the karyotype is also one of the only tests available for the evaluation of chromosomes in public health care. The state of Santa Catarina provides also CGH evaluation. Unfortunately, the availability of performing karyotype within the Brazilian National public Health System is far below the needs of the Brazilian population.

In this context, the aim of this study was to review the literature on the relationship of congenital heart defects with chromosomal abnormalities detected by the karyotype test.

## Method

Several researchers evaluated, in different studies, the presence of chromosomal abnormalities detected by karyotyping in patients with congenital heart disease. Therefore, we conducted a review of the scientific articles in MEDLINE database, using the descriptors: "*karyotype*" OR "*chromosomal*" OR "*chromosome*" AND "*heart defects*, *congenital*". The review encompassed both retrospective and prospective studies, in which all participants had congenital heart disease. In these, there should not be a selection regarding the type of heart defect, i.e., they should involve only cardiac malformations in general. Regarding the age range of participants, only studies involving children and adolescents were included. The research was delimited to articles published in English from 1980. Older studies, conducted before this decade, present important limitations, as they were developed in a time when the evaluation of chromosomes by banding and high-resolution techniques was still inexistent. Case reports, small case series or reviews, as well as studies conducted in the prenatal period were also excluded. Once we found different studies with the same sample, we chose to include only the main study.

With the use of descriptors in MEDLINE, 2,079 scientific articles were obtained. After applying the exclusion criteria (language other than English, case reports, small case series, reviews, publications before 1980, selected samples of congenital heart disease, and studies developed during the prenatal period), there were only 13 articles.

## Results and discussion

### Studies that assessed the frequency of chromosomal abnormalities identified through karyotype test in patients with congenital heart disease 

According to Table 1 ^(^ 16 ^,^ 19 ^,^ 29 ^,^ 33 ^-^ 42 ^)^, there was no study that assessed all patients of the same way. They were characterized by being, most of them, retrospective, developed in Europe, and not all patients of the samples studied underwent karyotype examination (most of the studies does not describe how many patients were assessed through this exam). We obtained only one study conducted in Latin America, in Brazil^(^ 29 ^)^. The sample sizes of the studies are also variable, being smaller on those developed prospectively^(^ 29 ^,^ 36 ^)^. 

**Table 1:**
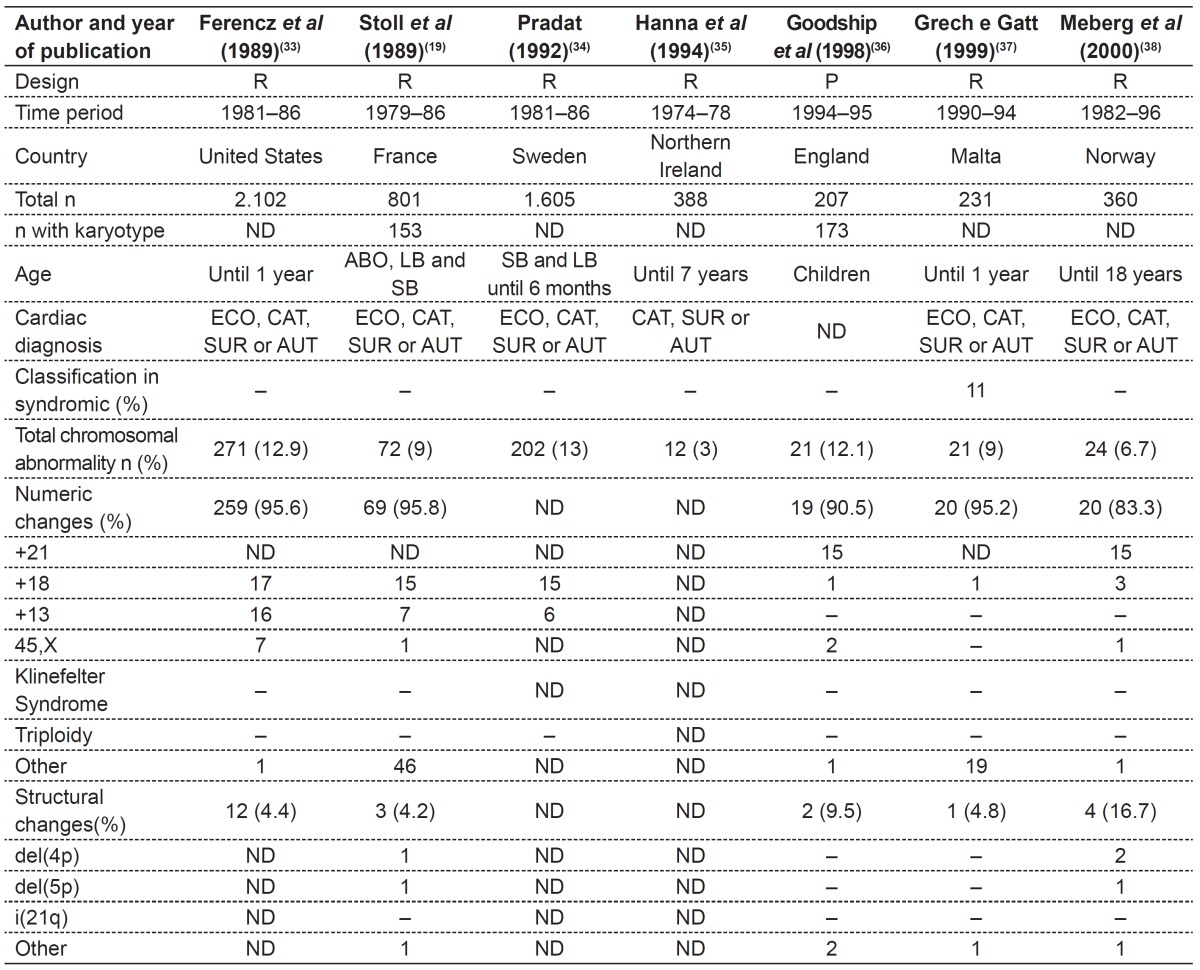

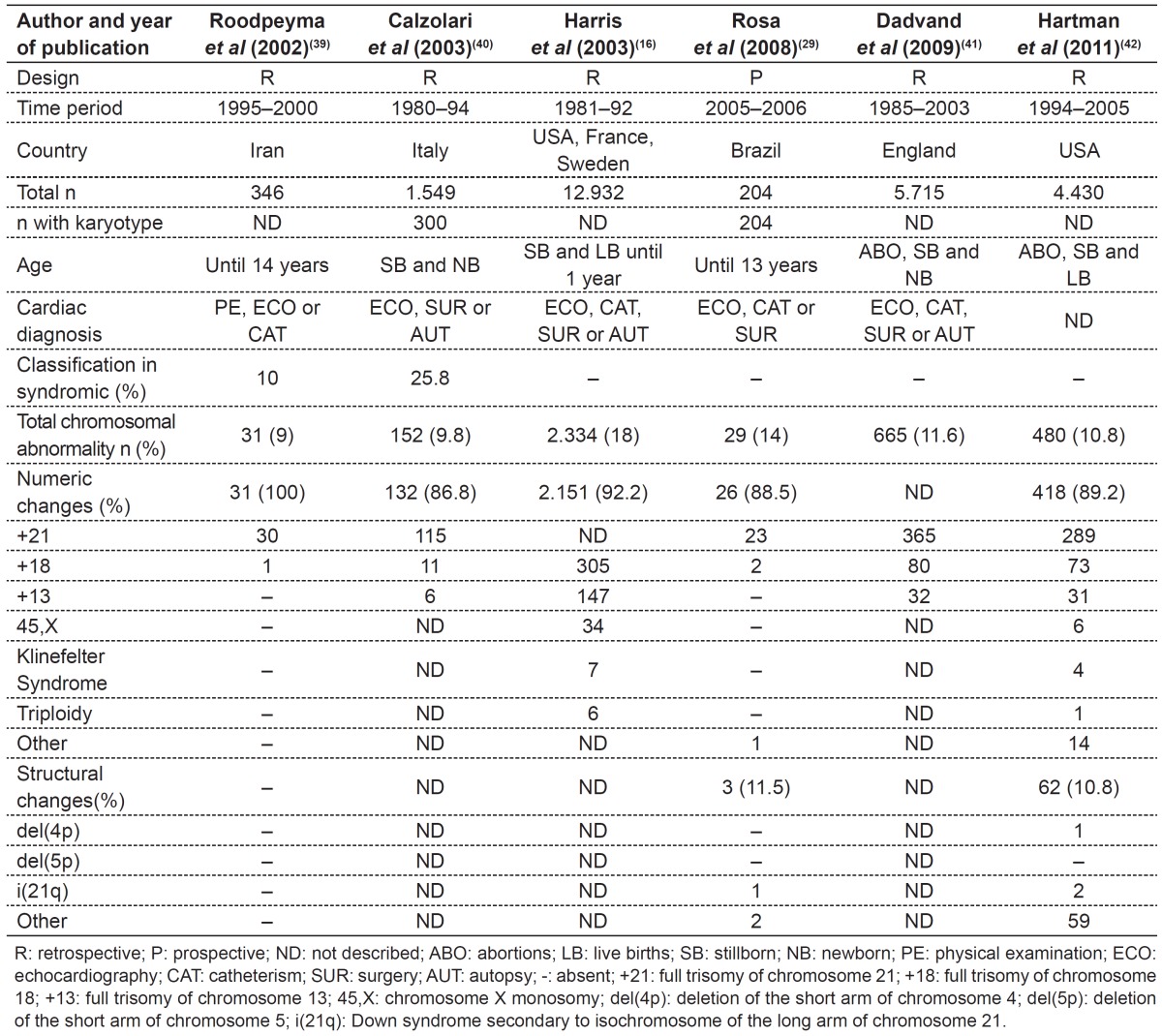
Comparison between different studies described in the literature

The age of analyzed patients also varied greatly. Some studies included spontaneous abortions and stillbirths. The top age limit observed was 18 years^(^ 38 ^)^. As for cardiac evaluation of patients, in most studies, there was a report of echocardiography, cardiac catheterization, surgery, and autopsy. Despite the classification of syndromic or not observed in some studies, there was no data describing the performance of dysmorphologic physical examination by a clinical geneticist. The classification of patients in syndromic ranged from 10 to 25.8% of the samples analyzed^(16,19,29,33-42) ^(Table 1). 

**Figure 1:**
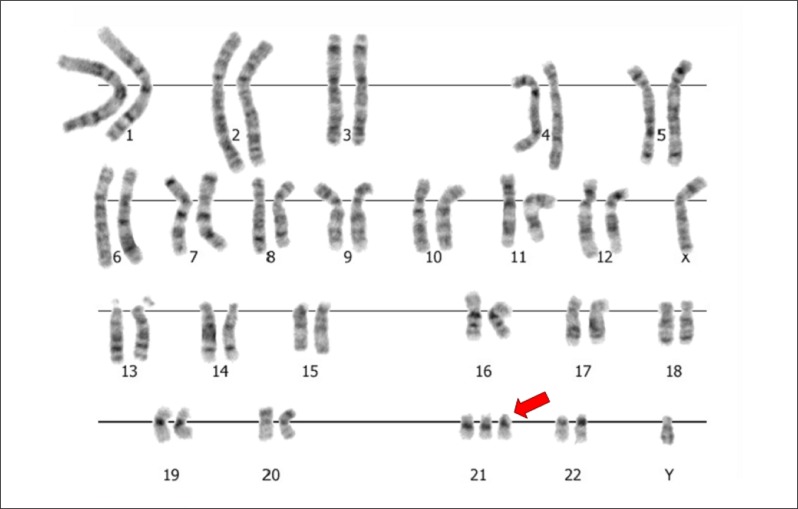
karyotype by GTG-banding (trypsin-Giemsa G-band) showing full trisomy of chromosome 21, compatible with Down syndrome. This is the main chromosomal abnormality observed in patients with congenital heart disease

The frequency of chromosome abnormalities detected by karyotype in patients with congenital heart disease ranged from 3 to 23% (usually around 9%)^(16,19,29,33-42) ^(Table 1). Thus, they are present in about one in every 10 patients with congenital heart disease, i.e., their frequency is about 12 times greater among individuals with congenital heart disease than in the general population, for which the rate is one in every 120 newborns^(^ 18 ^)^. The major chromosomal changes observed are numeric and correspond to the additional presence or lack of a chromosome. These were the first genetic abnormalities to be described in patients with congenital heart disease^(^ 17 ^)^ and usually account for over 80% of the abnormalities observed^(^ 16 ^,^ 19 ^,^ 29 ^,^ 33 ^-^ 42 ^)^. Among them, stands out the full trisomy of chromosome 21 (+21), the main chromosome constitution observed in individuals with Down syndrome (Figure 1). Another relatively common change was the full trisomy of chromosome 18 (+18), responsible for Edwards syndrome. Recurrent, but less frequent abnormalities consisted of full trisomy 13 (Patau syndrome), chromosome X monosomy (Turner syndrome), Klinefelter syndrome, and the triploidy syndrome^(16,19,29,33-42) ^(Table 1).

**Figure 2:**
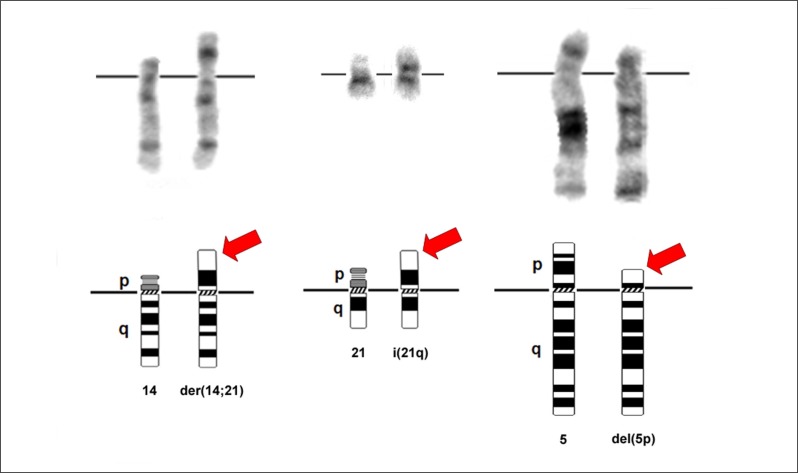
Partial karyotype by GTG-banding (trypsin-Giemsa G-band) and ideograms showing, respectively, a Robertsonian translocation between chromosomes 14 and 21 [der(14;21)], one isochromosome of the long arm (q) of chromosome 21 [i(21q)] and one partial interstitial deletion of long arm of chromosome 5 [del(5p)]. The first two forms represent changes associated with Down syndrome, and the third, to the Cri-du-Chat syndrome

A smaller percentage of chromosomal abnormalities observed in patients with congenital heart disease consisted of structural abnormalities. The main abnormalities correspond to those with loss (deletion) or gain (duplication) of part of a chromosome. Among them, stand out the deletion of the short arm of chromosome 4 (Wolf-Hirschhorn syndrome) and of chromosome 5 (Cri-du-Chat syndrome). The isochromosome of the long arm (chromosome with loss of short arm and duplication of the long arm) of chromosome 21, less common cause of Down syndrome, was also frequently described ^(16,19,29,33-42) ^(Table 1 and Figure 2). 

The chromosomal abnormalities most frequently observed and cited are characterized by having a high percentage of cardiac involvement. For instance, the frequency of congenital heart disease among individuals with Edwards and Patau syndrome ranges from 80 to 100%^(^ 43 ^-^ 46 ^)^. Furthermore, about 40 to 50% of patients with Down's syndrome have this defect^(16,17,20) ^(Table 2). Another important feature is the relationship of certain chromosomal abnormalities with specific heart defects. Down syndrome, for instance, shows association with atrioventricular septal defects^(^ 47 ^,^ 48 ^)^; and Edwards and Patau syndromes, with septal defects, such as interventricular and atrial communication. The polyvalvular disease is also common among these individuals^(^ 43 ^-^ 46 ^)^. Patients with Turner syndrome have more often bicuspid aortic valve and coarctation of the aorta^(^ 49 ^)^. The 22q11 deletion has great association with defects involving the outflow tract of the heart (conotruncal heart defects), such as interrupted aortic arch type B, *truncus arteriosus*, and the tetralogy of Fallot^(16,17,20,29-32) ^(Table 2). 

**Table 2:**
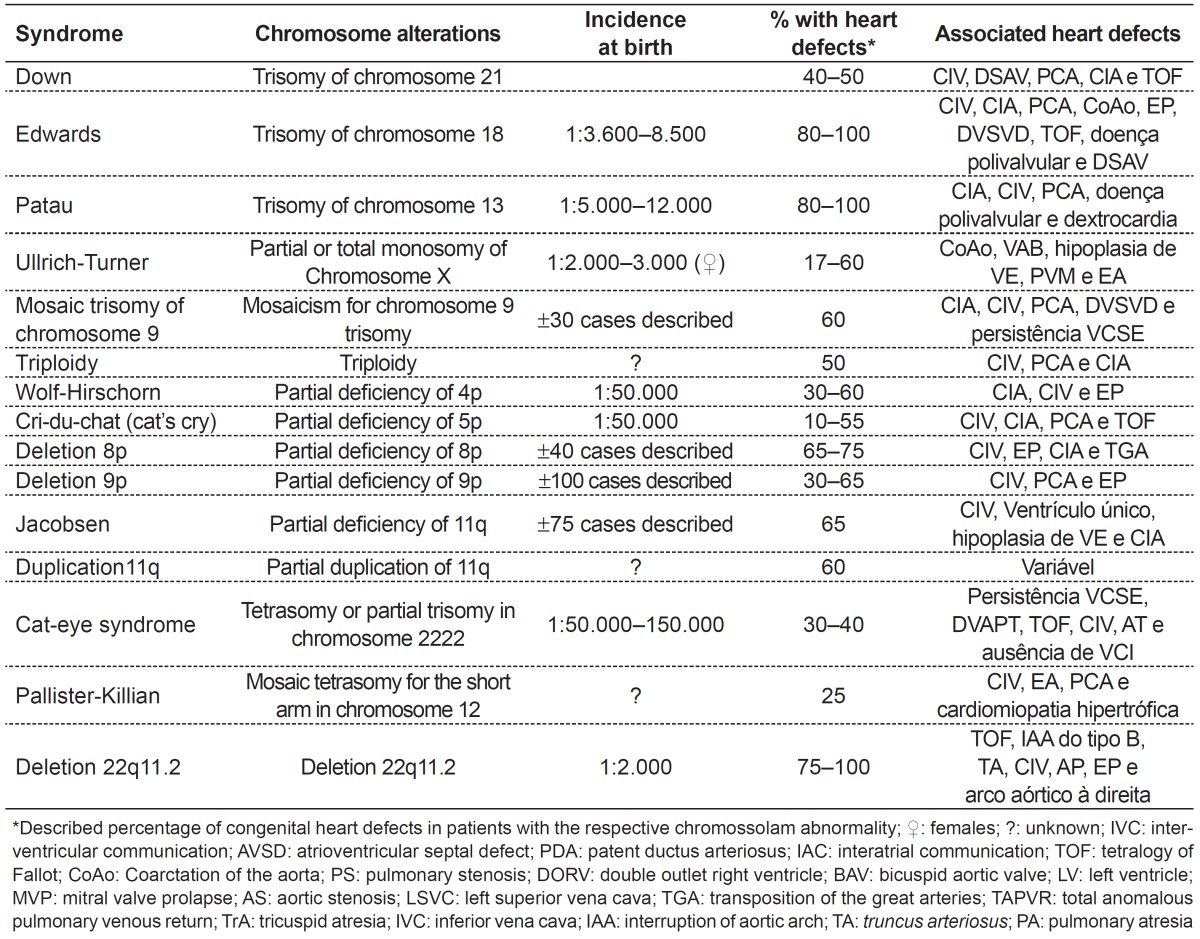
Main chromosomal abnormalities associated to cardiac malformations potentially detected through karyotype examination. Based on Marino and Digilio (2000)(20), Harris et al (2003)(16), and Fahed et al (2013)(17)

On the other hand, some types of heart defects showed a greater association with chromosomal abnormalities (Table 3)^(^ 50 ^)^. Among them, we highlight the atrioventricular septal defect (frequency greater than 50%, mainly due to Down syndrome), as well as interrupted aortic arch type B, *truncus arteriosus*, and tetralogy of Fallot (as already mentioned, they are rather associated with 22q11 deletion) (Table 3)^(^ 50 ^)^. The involvement of some chromosomal regions due to deletions and duplications is well reported in the literature, according to Table 4 ^(^ 51 ^)^.

**Table 3:**
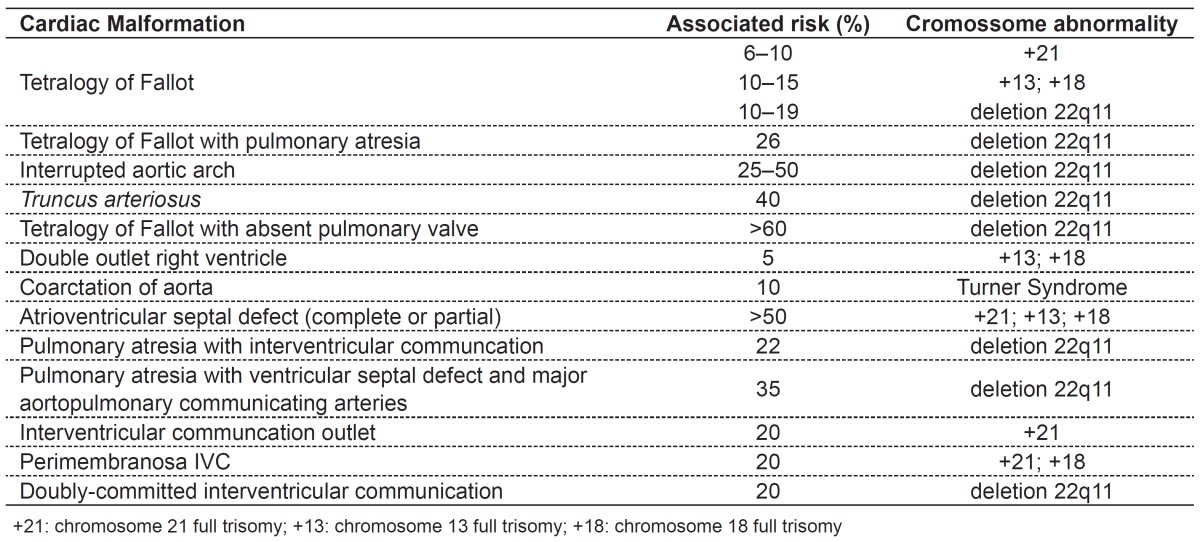
Cardiac malformations and their association with chromosomal abnormalities. Adapted from Manning et al(50)

**Table 4:**
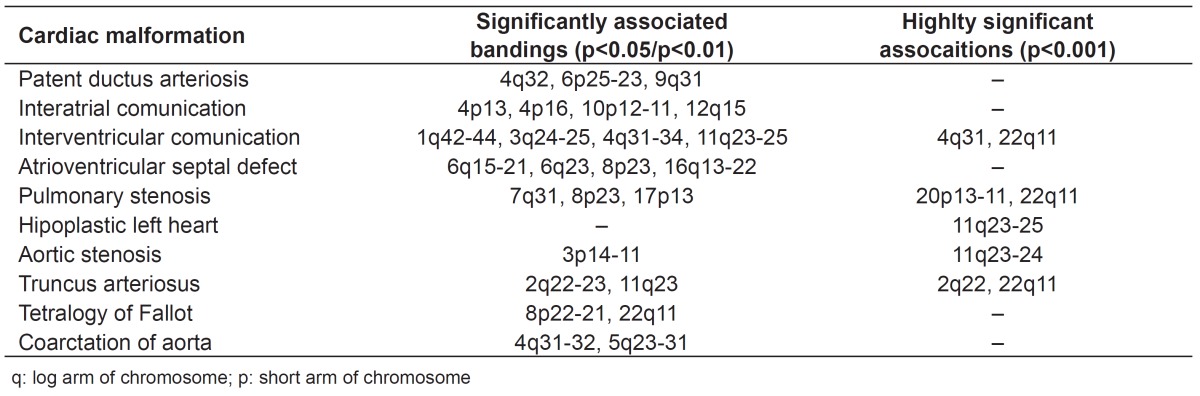
Regions of chromosome deletion statistically significant associated to specific heart malformations. Adapted from Brewer et al(51)

### Importance of identifying chromosomal abnormalities in patients with congenital heart disease 

As already mentioned, around 15 to 20% of patients with congenital heart disease present known etiology, and chromosomal abnormalities identified by karyotype stand out^(^ 5 ^,^ 18 ^)^. These are common in individuals with congenital heart disease, with a frequency of 3 to 23%, which highlights the importance of karyotyping for this population^(^ 16 ^,^ 19 ^,^ 29 ^,^ 33 ^-^ 42 ^)^.

Individuals with chromosomal disorders usually have an aspect that is considered syndromic, i.e., they present a scenario of dysmorphias, both major and minor, associated with other disabilities (such as intellectual) and behavioral changes. These dysmorphic features can be identified through a physical examination (dysmorphologic examination) conducted by a trained professional, such as the clinical geneticist or even a pediatrician with experience. Therefore, this professional is vital for the best choice of individuals to undergo genetic evaluation by examining the karyotype. 

Patients with chromosomal abnormalities often have associated extracardiac malformations, and are therefore at a higher risk of morbidity and mortality, which makes the cardiac surgery even riskier^(^ 20 ^,^ 35 ^,^ 52 ^,^ 53 ^)^. Moreover, such patients may require medical or surgical intensive procedures regardless of the heart disease^(^ 37 ^)^. Thus, in these cases, there is usually need for multidisciplinary assessment and monitoring, involving not only areas of Cardiology and Medical Genetics. It is also worth noting that some chromosomal abnormalities such as trisomy 13 (Patau syndrome) and trisomy 18 (Edwards syndrome), are associated with a very poor prognosis, and the literature discusses whether patients might actually benefit from heart surgery^(^ 44 ^,^ 45 ^)^. Therefore, all this information is critical for the patient's proper management and risk/prognosis assessment.

The importance of establishing an accurate diagnosis of the etiology of congenital heart disease also lies in the fact that families need genetic counseling with accurate information about the risks of recurrence^(^ 21 ^)^. Older studies on the recurrence of congenital heart disease suggested multifactorial inheritance^(^ 20 ^)^, because they simply measured familial aggregation and did not distinguish between genetic and non-genetic factors that could contribute to an increased risk to family members. In the case of chromosomal abnormalities, identification and definition are extremely important, because, depending on the abnormality observed, there may also be the need for assessment of other family members and a higher recurrence risk in the offspring. In cases of numerical abnormalities by full trisomy or total monosomy of a chromosome, there is no indication of parental karyotype assessment, because those are usually due to errors that occurred during gametogenesis. On the other hand, in cases of structural abnormalities, such as deletions and duplications, there is always an indication of parental karyotype, in order to rule out the hypothesis of one of them carrying a balanced chromosomal abnormality related to that observed in the child^(^ 18 ^)^.

It is worth noting, however, that the result of a traditional karyotype test does not exclude the fact that the patient might still present a syndrome. As shown previously, microscopic changes (such as microdeletions or microduplications) or gene mutations are not detected by this test. In such cases, clinical evaluation of the patient, especially by the geneticist, is essential to generate hypotheses and therefore choose appropriate tests for diagnosis.

Based on this review, authors believe that an accurate dysmorphologic examination, performed by an experienced pediatrician or by a geneticist, is rather important to indicate the karyotype in patients with congenital heart disease. This would help both to save costs with the exam and to the early identification of patients with chromosomal abnormalities, which might reflect in better supervision and genetic counseling.

## References

[1] Robinson A, Linden MG (1994). Clinical genetics handbook.

[2] Amorim LF, Pires CA, Lana AM, Campos AS, Aguiar RA, Tibúrcio JD (2008). Presentation of congenital heart disease diagnosed at
birth: analysis of 29,770 newborn infants. J Pediatr (Rio J).

[3] Kramer HH, Majewski F, Trampisch HJ, Rammos S, Bourgeois M (1987). Malformation patterns in children with congenital heart
disease. Am J Dis Child.

[4] Hoyme HE (1993). Minor anomalies: diagnostic clues to aberrant human
morphogenesis. Genetica.

[5]  Van der Bom T, Zomer AC, Zwinderman AH, Meijboom FJ, Bouma BJ, Mulder BJ (2011). The changing epidemiology of congenital heart
disease. Nat Rev Cardiol.

[6] Hoffman JI, Kaplan S (2002). The incidence of congenital heart
disease. J Am Coll Cardiol.

[7] Pinto VC, Daher CV, Sallum FS, Jatene MB, Croti UA (2004). The situation of congenital heart surgeries in
Brazil. Braz J Cardiovasc Surg.

[8] Ransom J, Srivastava D (2007). The genetics of cardiac birth defects. Semin Cell Dev Biol.

[9] Guitti JC (2000). Epidemiological characteristics of congenital heart
diseases in Londrina, Paraná south Brazil. Arq Bras Cardiol.

[10] Hagemann LL, Zielinsky P (2004). Rastreamento populacional de anormalidades cardíacas
fetais por ecocardiografia pré-natal em gestações de baixo risco no município de
Porto Alegre. Arq Bras Cardiol.

[11] Acharya G, Sitras V, Maltau JM, Dahl LB, Kaaresen PI, Hanssen TA (2004). Major congenital heart disease in Northern Norway:
shortcomings of pre- and postnatal diagnosis. Acta Obstet Gynecol Scand.

[12] Jenkins KJ, Correa A, Feinstein JA, Botto L, Britt AE, Daniels SR (2007). Noninherited risk factors and congenital cardiovascular
defects: current knowledge: a scientific statement from the American Heart
Association Council on Cardiovascular Disease in the Young: endorsed by the
American Academy of Pediatrics. Circulation.

[13] Pedra CA, Haddad J, Pedra SF, Peirone A, Pilla CB, Marin-Neto JA (2009). Paediatric and congenital heart disease in South
America: an overview. Heart.

[14] Dolk H, Loane M, Garne E, European Surveillance of Congenital Anomalies Working Group (2011). Congenital heart defects in Europe: prevalence and
perinatal mortality, 2000 to 2005. Circulation.

[15] Thulstrup AM, Bonde JP (2006). Maternal occupational exposure and risk of specific
birth defects. Occup Med (Lond).

[16] Harris JA, Francannet C, Pradat P, Robert E (2003). The epidemiology of cardiovascular defects, part 2: a
study based on data from three large registries of congenital
malformations. Pediatr Cardiol.

[17] Fahed AC, Gelb BD, Seidman JG, Seidman CE (2013). Genetics of congenital heart disease: the glass half
empty. Circ Res.

[18] Blue GM, Kirk EP, Sholler GF, Harvey RP, Winlaw DS (2012). Congenital heart disease: current knowledge about causes
and inheritance. Med J Aust.

[19] Stoll C, Alembik Y, Roth MP, Dott B, De Geeter B (1989). Risk factors in congenital heart disease. Eur J Epidemiol.

[20] Marino B, Digilio MC (2000). Congenital heart disease and genetic syndromes: specific
correlation between cardiac phenotype and genotype. Cardiovasc Pathol.

[21] Prasad C, Chudley AE (2002). Genetics and cardiac anomalies: the heart of the
matter. Indian J Pediatr.

[22] Tjio JH, Levan A (1956). The chromosome number of man. Hereditas.

[23] Lejeune J, Gautier M, Turpin R (1959). Étude des chromosomes somatiques de neuf enfants
mongoliens. C R Hebd Seances Acad Sci.

[24] Caspersson T, Zech L, Johansson C (1970). Differential binding of alkylating fluorochromes in
human chromosomes. Exp Cell Res.

[25] Yunis JJ (1981). New chromosomes techniques in the study of human
neoplasia. Hum Pathol.

[26] Smeets DF (2004). Historical prospective of human cytogenetics: from
microscope to microarray. Clin Biochem.

[27] Jauch A, Daumer C, Lichter P, Murken J, Schroeder-Kurth T, Cremer T (1990). Chromosomal in situ suppression hybridization of human
gonosomes and autosomes and its use in clinical cytogenetics. Hum Genet.

[28] Bejjani BA, Shaffer LG (2008). Clinical utility of contemporary molecular
cytogenetics. Annu Rev Genomics Hum Genet.

[29] Rosa RF, Pilla CB, Pereira VL, Flores JA, Golendziner E, Koshiyama DB (2008). 22q11.2 deletion syndrome in patients admitted to a
cardiac pediatric intensive care unit in Brazil. Am J Med Genet A.

[30] Rosa RF, Zen PR, Roman T, Graziadio C, Paskulin GA (2009). 22q11.2 deletion syndrome: catching the
CATCH22. Rev Paul Pediatr.

[31] Rosa RF, Trevisan P, Koshiyama DB, Pilla CB, Zen PR, Varella-Garcia M (2011). 22q11.2 deletion syndrome and complex congenital heart
defects. Rev Assoc Med Bras.

[32] Rosa RFM, Zen PRG, Graziadio C, Paskulin GA (2011). Síndrome de deleção 22q11.2 e cardiopatias
congênitas. Rev Paul Pediatr.

[33] Ferencz C, Neill CA, Boughman JA, Rubin JD, Brenner JI, Perry LW (1989). Congenital cardiovascular malformations associated with
chromosome abnormalities: an epidemiologic study. J Pediatr.

[34] Pradat P (1992). Epidemiology of major congenital heart defects in
Sweden, 1981-1986. J Epidemiol Community Health.

[35] Hanna EF, Nevin NC, Nelson J (1994). Genetic study of congenital heart defects in Northern
Ireland (1974-1978). J Med Genet.

[36] Goodship J, Cross I, Liling J, Wren C (1998). A population study of chromosome 22q11 deletions in
infancy. Arch Dis Child.

[37] Grech V, Gatt M (1999). Syndromes and malformations associated with congenital
heart disease in a population-based study. Int J Cardiol.

[38] Meberg A, Otterstad JE, Frøland G, Lindberg H, Sørland SJ (2000). Outcome of congenital heart defects - a population-based
study. Acta Paediatr.

[39] Roodpeyma S, Kamali Z, Afshar F, Naraghi S (2002). Risk factors in congenital heart disease. Clin Pediatr.

[40] Calzolari E, Garani G, Cocchi G, Magnani C, Rivieri F, Neville A (2003). Congenital heart defects: 15 years of experience of the
Emilia-Romagna Registry (Italy). Eur J Epidemiol.

[41] Dadvand P, Rankin J, Shirley MD, Rushton S, Pless-Mulloli T (2009). Descriptive epidemiology of congenital heart disease in
Northern England. Paediatr Perinat Epidemiol.

[42] Hartman RJ, Rasmussen SA, Botto LD, Riehle-Colarusso T, Martin CL, Cragan JD (2011). The contribution of chromosomal abnormalities to
congenital heart defects: a population-based study. Pediatr Cardiol.

[43] Rosa RF, Rosa RC, Lorenzen MB, de Moraes FN, Graziadio C, Zen PR (2011). Trisomy 18: experience of a reference hospital from the
south of Brazil. Am J Med Genet A.

[44] Rosa RF, Rosa RC, Lorenzen MB, de Oliveira CA, Graziadio C, Zen PR (2012). Trisomy 18: frequency, types, and prognosis of
congenital heart defects in a Brazilian cohort. Am J Med Genet A.

[45] Petry P, Polli JB, Mattos VF, Rosa RC, Zen PR, Graziadio C (2013). Clinical features and prognosis of a sample of patients
with trisomy 13 (Patau syndrome) from Brazil. Am J Med Genet A.

[46] Rosa RF, Rosa RC, Zen PR, Graziadio C, Paskulin GA (2013). Trisomy 18: review of the clinical, etiologic,
prognostic, and ethical aspects. Rev Paul Pediatr.

[47] Nisli K (2009). Prevalence of congenital heart defects in patients with
Down's syndrome. J Pediatr (Rio J).

[48] Vilas Boas LT, Albernaz EP, Costa RG (2009). Prevalence of congenital heart defects in patients with
Down syndrome in the municipality of Pelotas, Brazil. J Pediatr (Rio J).

[49] Carvalho AB, Guerra G, Baptista MT, de Faria AP, Marini SH, Guerra AT (2010). Cardiovascular and renal anomalies in Turner
syndrome. Rev Assoc Med Bras.

[50] Manning N, Kaufman L, Roberts P (2005). Genetics of cardiological disorders. Semin Fetal Neonatal Med.

[51] Brewer C, Holloway S, Zawalnyski P, Schinzel A, FitzPatrick D (1998). A chromosomal deletion map of human
malformations. Am J Hum Genet.

[52] Begic H, Tahirovic H, Mesihovic-Dinarevic S, Ferkovic V, Atic N, Latifagic A (2003). Epidemiological and clinical aspects of congenital heart
disease in children in Tuzla Canton, Bosnia-Herzegovina. Eur J Pediatr.

[53] Gonzalez JH, Shirali GS, Atz AM, Taylor SN, Forbus GA, Zyblewski SC (2009). Universal screening for extracardiac abnormalities in
neonates with congenital heart disease. Pediatr Cardiol.

