# Supplementary material for: Congenital heart disease and chromossomopathies detected by the karyotype
Source: Rev Paul Pediatr. 2014 Jun;32(2):262–71. doi: 10.1590/0103-0582201432213213 (PMC4183026; doi:10.1590/0103-0582201432213213)
Supplement: Translation

# Translation: Cardiopatias congênitas e cromossomopatias detectadas por meio do cariótipo

**Authors:** Patrícia Trevisan, Rafael Fabiano M. Rosa, Dayane Bohn Koshiyama, Tatiana Diehl Zen, Giorgio Adriano Paskulin, Paulo Ricardo G. Zen

**Affiliations:** 1 UFCSPA, Porto Alegre, RS, Brasil; 2 Hospital Materno Infantil Presidente Vargas (HMIPV), Porto Alegre, RS, Brasil

**Keywords:** cardiopatias congênitas, cariótipo, síndrome de Down, trissomia, aberrações cromossômicas

## Abstract

**OBJETIVO:**

Realizar uma revisão da literatura sobre a relação das cardiopatias congênitas com anormalidades cromossômicas detectadas por meio do exame de cariótipo.

**FONTES DE DADOS:**

Pesquisaram-se artigos científicos no portal MEDLINE, utilizando-se os descritores "*karyotype*" OR "*chromosomal*" OR "*chromosome*" AND "*heart defects*, *congenital*". A pesquisa limitou-se a artigos publicados em inglês a partir da década de 1980.

**SÍNTESE DOS DADOS:**

As cardiopatias congênitas são um grupo de lesões etiologicamente heterogêneo e pouco compreendido. Vários pesquisadores avaliaram a presença de anormalidades cromossômicas detectadas pelo exame de cariótipo em pacientes portadores de cardiopatia congênita. Porém, a maioria dos artigos era composta de trabalhos retrospectivos desenvolvidos na Europa, nos quais nem todos os pacientes foram submetidos à avaliação cariotípica. Nesta revisão, encontrou-se apenas um estudo desenvolvido na América Latina, no Brasil. Sabe-se que anormalidades cromossômicas são frequentes, estando presentes em cerca de um em cada dez pacientes com cardiopatia congênita. Dentre as alterações observadas, destaca-se a trissomia do cromossomo 21 (síndrome de Down). Esses pacientes frequentemente apresentam malformações extracardíacas associadas e risco maior de morbidade e mortalidade, tornando a cirurgia cardíaca ainda mais arriscada.

**CONCLUSÕES:**

Apesar de todos os avanços ocorridos nas últimas décadas na área da citogenética, o exame de cariótipo continua sendo uma ferramenta fundamental para se avaliarem pacientes com cardiopatia congênita. O exame físico dismorfológico minucioso é de grande importância para indicar a realização do cariótipo.

## Introdução

As malformações congênitas são detectadas em aproximadamente 3 a 5% dos recém-nascidos^(^ ^1^ ^)^, sendo que um em cada 33 possui anormalidades graves^(^ ^2^ ^)^. Malformações maiores são aquelas que causam efeito adverso na aceitabilidade social do indivíduo ou na função de um determinado órgão ou sistema^(^ ^3^ ^)^. Por outro lado, malformações menores não apresentam significado estético ou funcional para o indivíduo afetado, sendo esse um achado estrutural visto em menos de 4% da população geral. Contudo, algumas anomalias menores podem ser marcadoras externas de anomalias maiores específicas, eventualmente ocultas. Dessa maneira, a maioria das síndromes poderia ser reconhecida pelo geneticista clínico se fossem levados em consideração esses padrões de anomalias menores. O exame dismorfológico, portanto, poderia auxiliar na decisão de se realizar investigação complementar, como, por exemplo, por meio do cariótipo^(^ ^4^ ^)^. 

Dentre as malformações congênitas mais frequentes, destacam-se as cardiopatias congênitas, compostas por alterações estruturais e funcionais do coração presentes ao nascer, independentemente do momento do diagnóstico. As cardiopatias congênitas são um grupo heterogêneo de lesões com consequências hemodinâmicas variadas, necessitando de diferentes seguimentos e intervenções^(^ ^5^ ^)^. Estudos demonstram que a incidência das cardiopatias congênitas pode variar de quatro a 14 por 1.000 nascidos vivos^(^ ^6^ ^-^ ^8^ ^)^. No Brasil, descreveu-se uma prevalência que varia de cinco a 12 por 1.000 nascidos vivos^(^ ^2^ ^,^ ^9^ ^,^ ^10^ ^)^. Essas variações podem ser explicadas por diversos fatores, como a ocorrência de defeitos letais que impossibilitam o nascimento vivo do concepto e a não inclusão de defeitos cardíacos menores. Estudos demonstram que a cardiopatia congênita pode ser responsável por cerca de 40% de todos os defeitos congênitos, sendo considerada uma das malformações mais frequentes^(^ ^11^ ^,^ ^12^ ^)^. No Brasil, apesar de sua grande extensão geográfica, dispõem-se de 12 centros especializados tanto no diagnóstico quanto no tratamento de pacientes com cardiopatia congênita^(^ ^13^ ^)^. A necessidade média de cirurgia cardiovascular em congênitos no Brasil é da ordem de 23.077 procedimentos por ano. Contudo, a rede de saúde instalada não é suficiente e, em 2002, por exemplo, houve um déficit de cirurgias que chegou a 65%^(^ ^7^ ^)^. 

Assim, as cardiopatias congênitas ainda representam um problema maior de Saúde Pública pelo mundo, sendo a principal causa de morte entre malformações congênitas^(^ ^12^ ^)^. Defeitos cardíacos graves e moderadamente graves perfazem cerca de três a seis em cada 1.000 nascidos vivos e caracterizam-se pela necessidade de cuidados intensivos e cirúrgicos mais complexos^(^ ^6^ ^,^ ^11^ ^,^ ^14^ ^)^. Esses defeitos representam uma importante causa de admissão e de mortalidade em unidades pediátricas de tratamento intensivo^(^ ^15^ ^)^. No Rio Grande do Sul, contudo, a maioria das unidades de tratamento intensivo encontra-se superlotada e, muitas vezes, não possui equipamentos e profissionais especializados para o diagnóstico diferencial, nem condições para o tratamento cirúrgico de pacientes portadores de cardiopatia congênita^(^ ^10^ ^)^. Isso pode dever-se ao fato de que países menos desenvolvidos possuem outras prioridades relacionadas à saúde, incluindo a prevenção da desnutrição e promoção de campanhas de vacinação^(^ ^13^ ^)^.

Sabe-se que o coração é o primeiro órgão a ser formado no embrião, sendo fundamental para o provimento de oxigênio e nutrientes para o desenvolvimento fetal^(^ ^8^ ^)^. Sua formação é complexa e ocorre durante diversas semanas da vida embrionária, tornando-o vulnerável à ocorrência de falhas no desenvolvimento^(^ ^15^ ^)^. As cardiopatias congênitas são consideradas malformações etiologicamente heterogêneas e pouco compreendidas^(^ ^16^ ^,^ ^17^ ^)^. Apenas cerca de 15-20% dos casos são atribuídos a causas conhecidas^(^ ^5^ ^,^ ^18^ ^)^ e, dentre elas, destacam-se as anormalidades cromossômicas^(^ ^17^ ^,^ ^19^ ^)^, que são mais frequentes em pacientes com cardiopatia congênita do que na população em geral^(^ ^16^ ^,^ ^20^ ^,^ ^21^ ^)^. 

Os primeiros passos para o desenvolvimento do cariótipo começaram a ser dados com o entendimento da ação da colchicina e do tratamento hipotônico sobre as células, ocorridos na primeira metade do século 20. A determinação do número correto de cromossomos humanos nas células somáticas (n=46) por Tjio e Levan, em 1959^(^ ^22^ ^)^, foi a base para se identificarem as síndromes cromossômicas. Em 1959, Lejeune *et al* descreveram a primeira trissomia de cromossomos autossômicos, em um caso de síndrome de Down^(^ ^23^ ^)^. Algumas décadas depois, a introdução das técnicas de coloração longitudinal dos cromossomos, conhecidas como "bandamento"^(^ ^24^ ^)^, e o surgimento das técnicas de alta resolução cromossômica^(^ ^25^ ^)^ fizeram com que as alterações cromossômicas numéricas e estruturais passassem a ser mais bem reconhecidas e diagnosticadas. Como visto pela sua descrição histórica, o cariótipo é um exame considerado quase artesanal, baseado no cultivo de células (usualmente do sangue), que possui inúmeras etapas e, por isso, é potencialmente sujeito a falhas (devido, por exemplo, à forma de coleta do material), além de os resultados serem obtidos algumas semanas depois da coleta do exame^(^ ^26^ ^)^.

 Com o desenvolvimento das sondas de DNA e das técnicas de hibridização *in situ* fluorescente (*fluorescence in situ hybridization* - FISH), cariotipagem espectral (SKY) e hibridização genômica comparativa (CGH), a partir da década de 1980, criou-se um novo conceito, o da citogenética molecular^(^ ^26^ ^,^ ^27^ ^)^. Essas novas técnicas possibilitaram a identificação de alterações complexas e sutis, como deleções e duplicações muito pequenas (microdeleções e microduplicações, respectivamente), que podem escapar à análise cromossômica padrão por meio do cariótipo. Outra vantagem em relação ao exame de cariótipo é que muitas delas podem não necessitar de cultivo celular para análise, agilizando os resultados^(^ ^26^ ^,^ ^28^ ^)^. Essas técnicas apresentam alto custo, superior ao do cariótipo, porém sua implementação permitiu a identificação de novas condições, como a síndrome de deleção 22q11, também conhecida como síndrome velocardiofacial ou DiGeorge, uma doença genética bastante relacionada às cardiopatias congênitas e que, na maioria das vezes, escapa à detecção por meio do cariótipo^(^ ^29^ ^-^ ^32^ ^)^. 

Contudo, apesar de todos os avanços, o cariótipo, mesmo com suas limitações, continua a ser uma ferramenta fundamental na avaliação genética dos pacientes, incluindo aqueles com cardiopatia congênita. O cariótipo se aplica principalmente àqueles pacientes que apresentam anomalias menores ou outras alterações maiores extracardíacas. Como visto, estas podem ser marcadoras de condições eventualmente ocultas, como algumas síndromes. Daí a importância da avaliação dismorfológica para melhor selecionar os casos a serem testados. Em nosso meio, o cariótipo também é um dos únicos exames de avaliação dos cromossomos disponível na rede pública de atendimento em saúde, o Sistema Único de Saúde (SUS). O Estado de Santa Catarina disponibiliza também a avaliação por meio da CGH. Infelizmente, a disponibilidade de realização de cariótipo no âmbito do SUS é muito aquém das necessidades da população brasileira.

Nesse contexto, o objetivo deste trabalho foi realizar uma revisão da literatura sobre a relação das cardiopatias congênitas com anormalidades cromossômicas detectadas por meio do exame de cariótipo.

## Método

Vários pesquisadores avaliaram, em diferentes estudos, a presença de anormalidades cromossômicas detectadas por meio do exame de cariótipo em pacientes portadores de cardiopatia congênita. Assim, realizou-se uma revisão dos artigos científicos presentes no portal MEDLINE, utilizando-se os descritores: "*karyotype*" OR "*chromosomal*" OR "*chromosome*" AND "*heart defects*, *congenital*". A revisão englobou estudos tanto retrospectivos quanto prospectivos, nos quais todos os participantes eram portadores de cardiopatia congênita. Nestes, não deveria haver seleção quanto ao tipo de defeito cardíaco, ou seja, deveriam envolver somente malformações cardíacas em geral. Quanto à faixa etária dos participantes, incluíram-se apenas estudos que envolveram crianças e adolescentes. A pesquisa delimitou-se a artigos publicados em inglês a partir da década de 1980. Estudos mais antigos, realizados antes dessa década, apresentam importantes limitações, pois foram desenvolvidos em uma época em que ainda não existia a avaliação dos cromossomos por técnicas de bandamento e de alta resolução e, por isso, foram excluídos da análise. Excluíram-se também relatos de caso, pequenas séries de caso ou revisões, bem como estudos desenvolvidos no período pré-natal. Diante de diferentes pesquisas desenvolvidas com a mesma amostra, optou-se por incluir apenas o estudo principal.

Com a utilização dos descritores no portal MEDLINE, obtiveram-se 2.079 artigos científicos. Após a aplicação dos critérios de exclusão (idioma diferente do inglês, relatos de caso, pequenas séries de caso, revisões, publicações antes de 1980, amostras selecionadas de cardiopatia congênita e estudos desenvolvidos no período pré-natal), restaram apenas 13 artigos.

## Estudos que avaliaram a frequência de anormalidades cromossômicas identificadas por meio do exame de cariótipo em pacientes com cardiopatia congênita

Conforme a Tabela 1 ^(^ ^16^ ^,^ ^19^ ^,^ ^29^ ^,^ ^33^ ^-^ ^42^ ^)^, não houve um estudo que avaliou todos os pacientes do mesmo modo. Estes se caracterizaram por serem, na sua maioria, retrospectivos, desenvolvidos na Europa e nem todos os pacientes das amostras estudadas foram submetidos à avaliação cariotípica (a maioria não descreve quantos pacientes foram avaliados por meio desse exame). Obteve-se apenas um estudo desenvolvido na América Latina, no caso, no Brasil^(^ ^29^ ^)^. Os tamanhos amostrais dos trabalhos também são variáveis, sendo menores naqueles desenvolvidos prospectivamente^(^ ^29^ ^,^ ^36^ ^)^. 

**Tabela 1:** Comparação entre os diferentes estudos descritos na literatura mundial

A idade dos pacientes em avaliação também variou bastante. Alguns estudos incluíram abortamentos espontâneos e natimortos. O limite superior de idade observado foi de 18 anos^(^ ^38^ ^)^. Quanto à avaliação cardiológica dos pacientes, na maioria dos trabalhos, houve a descrição de ecocardiografia, cateterismo cardíaco, cirurgia e autópsia. Apesar da classificação de sindrômico ou não observada em alguns trabalhos, não foram recuperados dados que descrevessem a realização de exame físico dismorfológico por um geneticista clínico. A classificação dos pacientes em sindrômicos variou de 10 a 25,8% das amostras analisadas^(16,19,29,33-42) ^(Tabela 1). 

A frequência de anormalidades cromossômicas detectadas por meio do cariótipo em pacientes com cardiopatia congênita variou de 3 a 23% (em geral, em torno de 9%)^(16,19,29,33-42) ^(Tabela 1). Assim, elas estão presentes em cerca de um em cada 10 pacientes com cardiopatia congênita, ou seja, sua frequência é cerca de 12 vezes maior entre indivíduos com cardiopatia congênita do que na população em geral, na qual a frequência é de em um em cada 120 recém-nascidos^(^ ^18^ ^)^. As principais alterações cromossômicas observadas são numéricas e correspondem à presença adicional ou à falta de um cromossomo. Estas foram as primeiras anormalidades genéticas a serem descritas em pacientes com cardiopatia congênita^(^ ^17^ ^)^ e usualmente correspondem a mais de 80% das anormalidades observadas^(^ ^16^ ^,^ ^19^ ^,^ ^29^ ^,^ ^33^ ^-^ ^42^ ^)^. Dentre elas, destaca-se a trissomia livre do cromossomo 21 (+21), a principal constituição cromossômica observada em indivíduos com síndrome de Down (Figura 1). Outra alteração relativamente comum foi a trissomia livre do cromossomo 18 (+18), responsável pela síndrome de Edwards. Anormalidades recorrentes, mas menos frequentes, consistiram da trissomia livre do cromossomo 13 (síndrome de Patau), da monossomia do cromossomo X (síndrome de Turner), da síndrome de Klinefelter e da triploidia^(16,19,29,33-42) ^(Tabela 1).

**Figura 1:** Cariótipo por bandas GTG (banda G por tripsina e Giemsa) evidenciando trissomia livre do cromossomo 21, compatível com síndrome de Down. Essa é a principal anormalidade cromossômica observada entre pacientes com cardiopatia congênita

Um percentual menor das alterações cromossômicas verificadas em pacientes com cardiopatia congênita consistiu de anormalidades estruturais. As principais anormalidades correspondem àquelas com perda (deleção) ou ganho (duplicação) de parte de um cromossomo. Dentre elas, destacam-se a deleção do braço curto do cromossomo 4 (síndrome de Wolf-Hirschhorn) e do cromossomo 5 (síndrome do Cri-du-Chat). O isocromossomo do braço longo (cromossomo com perda do braço curto e duplicação do longo) do cromossomo 21, causa menos comum de síndrome de Down, também foi frequentemente descrito^(16,19,29,33-42) ^(Tabela 1 e Figura 2). 

**Figura 2:** Cariótipo parcial por bandas GTG (banda G por tripsina e Giemsa) e ideogramas evidenciando, respectivamente, uma translocação Robertsoniana entre os cromossomos 14 e 21 [der(14;21)], um isocromossomo do braço longo (q) do cromossomo 21 [i(21q)] e uma deleção parcial e intersticial do braço curto do cromossomo 5 [del(5p)]. As duas primeiras alterações representam formas associadas à síndrome de Down e a terceira, à síndrome do Cri-du-Chat

As anormalidades cromossômicas mais frequentemente observadas e citadas caracterizam-se por apresentar alto percentual de acometimento cardíaco. Por exemplo, a frequência de cardiopatias congênitas entre indivíduos com síndrome de Edwards e de Patau varia de 80 a 100%^(^ ^43^ ^-^ ^46^ ^)^. Além disso, cerca de 40 a 50% dos pacientes com síndrome de Down possuem essa malformação^(16,17,20) ^(Tabela 2). Outra característica importante é a relação de certas anormalidades cromossômicas com defeitos cardíacos específicos. A síndrome de Down, por exemplo, apresenta associação com defeitos do septo atrioventricular^(^ ^47^ ^,^ ^48^ ^)^; as síndromes de Edwards e de Patau, com defeitos septais, como a comunicação interventricular e a comunicação atrial. A doença polivalvular é também comum entre esses indivíduos^(^ ^43^ ^-^ ^46^ ^)^. Pacientes com síndrome de Turner apresentam mais frequentemente válvula aórtica bicúspide e coarctação de aorta^(^ ^49^ ^)^. A deleção 22q11 possui grande associação com defeitos que envolvem as vias de saída do coração (conotruncais), como a interrupção do arco aórtico do tipo B, o *truncus arteriosus* e a tetralogia de Fallot^(16,17,20,29-32) ^(Tabela 2). 

**Tabela 2:** Principais anormalidades cromossômicas associadas com malformações cardíacas potencialmente detectadas por meio do exame de cariótipo

Por outro lado, alguns tipos de defeitos cardíacos apresentaram maior associação com anormalidades cromossômicas (Tabela 3)^(^ ^50^ ^)^. Dentre eles, destacam-se o defeito de septo atrioventricular (frequência superior a 50%, devido, principalmente, à síndrome de Down), bem como a interrupção do arco aórtico do tipo B, o *truncus arteriosus* e a tetralogia de Fallot (como já citado, são bastante associados à deleção 22q11) (Tabela 3)^(^ ^50^ ^)^. O acometimento de algumas regiões cromossômicas em decorrência de deleções e duplicações é bem relatado na literatura, conforme a Tabela 4 ^(^ ^51^ ^)^.

**Tabela 3:** Malformações cardíacas e suas associações com alterações cromossômicas

**Tabela 4:** Regiões de deleção cromossômica estatisticamente significantes associadas com malformações cardíacas congênitas específicas.

## Importância da identificação de anormalidades cromossômicas em pacientes com cardiopatia congênita

Como visto, cerca de 15 a 20% dos pacientes com cardiopatias congênitas apresentam etiologia conhecida, destacando-se as alterações cromossômicas identificadas por meio do cariótipo^(^ ^5^ ^,^ ^18^ ^)^. Estas são comuns em indivíduos portadores de cardiopatia congênita, com frequência de 3 a 23%, o que salienta a importância do cariótipo para essa população^(^ ^16^ ^,^ ^19^ ^,^ ^29^ ^,^ ^33^ ^-^ ^42^ ^)^.

Indivíduos portadores de cromossomopatia usualmente apresentam um aspecto considerado sindrômico, ou seja, possuem um quadro de dismorfias, tanto menores quanto maiores, associado a outras deficiências (como intelectual) e alterações comportamentais. Essas dismorfias podem ser identificadas por meio de um exame físico (exame dismorfológico) realizado por um profissional treinado, como o geneticista clínico ou mesmo um pediatra com experiência. Assim, esse profissional é fundamental para a melhor escolha dos indivíduos a serem submetidos à avaliação genética por meio do exame de cariótipo. 

Pacientes portadores de anormalidade cromossômica frequentemente apresentam malformações extracardíacas associadas, possuindo, assim, maior risco de morbidade e de mortalidade, o que torna a cirurgia cardíaca mais arriscada^(^ ^20^ ^,^ ^35^ ^,^ ^52^ ^,^ ^53^ ^)^. Além disso, tais pacientes podem necessitar de procedimento cirúrgico ou intensivo clínico independentemente da doença cardíaca^(^ ^37^ ^)^. Assim, nesses casos, usualmente há necessidade de avaliação e acompanhamento multidisciplinar, envolvendo não só as áreas da Cardiologia e da Genética Médica. Cabe ressaltar também que algumas anormalidades cromossômicas, como a trissomia do cromossomo 13 (síndrome de Patau) e a trissomia do cromossomo 18 (síndrome de Edwards), são associadas a um prognóstico bastante grave, discutindo-se na literatura se tais pacientes poderiam de fato se beneficiar do tratamento cirúrgico do coração^(^ ^44^ ^,^ ^45^ ^)^. Assim, todas essas informações são fundamentais para o adequado manejo e avaliação de riscos/prognóstico dos pacientes.

A importância de se estabelecer um diagnóstico preciso para a etiologia da cardiopatia congênita reside também no fato de que as famílias necessitam de aconselhamento genético com informação acurada sobre os riscos de recorrência^(^ ^21^ ^)^. Estudos mais antigos sobre a recorrência da doença cardíaca congênita sugeriam herança multifatorial^(^ ^20^ ^)^, pois simplesmente mensuravam a agregação familiar e não distinguiam entre os fatores genéticos e não genéticos que poderiam contribuir para um risco aumentado aos familiares. No caso das alterações cromossômicas, sua identificação e definição são extremamente importantes, pois, dependendo do tipo de anormalidade observada, pode haver necessidade de avaliação também de outros membros da família e um risco aumentado de recorrência para a prole. Nos casos de anormalidades numéricas por trissomia livre ou mesmo monossomia total de um cromossomo, não existe indicação de avaliação cariotípica dos pais, pois usualmente são decorrentes de erros ocorridos durante a gametogênese. Por outro lado, nos casos de anormalidades estruturais, como deleções e duplicações, sempre há indicação de cariótipo dos pais, a fim de excluir a hipótese de algum deles ser portador de alteração cromossômica equilibrada relacionada àquela observada na criança^(^ ^18^ ^)^.

Cabe ressaltar, contudo, que o resultado de um exame de cariótipo normal não exclui o fato de que o paciente ainda pode apresentar uma síndrome. Como mostrado anteriormente, alterações microscópicas (como microdeleções ou microduplicações) ou mutações gênicas não são detectadas por meio desse exame. Nesses casos, a avaliação clínica do paciente, especialmente pelo médico geneticista, é fundamental para gerar hipóteses e, consequentemente, escolher os exames apropriados para o diagnóstico.

Com base nesta revisão, os autores acreditam que o exame físico dismorfológico minucioso, realizado por um pediatra experiente ou por um geneticista, seja muito importante para indicar a realização do cariótipo em pacientes com cardiopatia congênita. Isso ajudaria tanto a poupar custos com a realização do exame quanto a identificar precocemente pacientes portadores de anormalidades cromossômicas, o que poderia se refletir em melhor manejo e aconselhamento genético.

## Agradecimentos

Agradecemos à Coordenação de Aperfeiçoamento de Pessoal de Nível Superior (CAPES) pela bolsa de estudos recebida.
